# Superior Optic Vein Thrombosis Related to Orbital Cellulitis Secondary to Aquatic Injury

**DOI:** 10.7759/cureus.7080

**Published:** 2020-02-23

**Authors:** Zeynep Günes Ozunal, Sibel Karsidag, Sevki Sahin

**Affiliations:** 1 Medical Pharmacology, Maltepe University, İstanbul, TUR; 2 Neurology, Maltepe University, İstanbul, TUR

**Keywords:** pharmacotherapy, thrombosis, cellulitis

## Abstract

A 52-year-old woman presented with orbital cellulitis and sixth cranial nerve palsy as a result of striking the tail of a stingray while swimming. Her ophthalmologic and neurologic examination showed injury of the conjunctiva, corneal abrasion without mention of foreign body, contusion of the eyelid, and isolated lateral gaze palsy and ptosis in the right eye. Orbital magnetic resonance (MR) imaging and MR venography showed orbital cellulitis, superior and lateral rectus edema, and thrombosis of the superior ophthalmic vein on the right eye. She was treated appropriately, and her physical examination showed significant improvement within three months.

## Introduction

Stingrays are cartilaginous fish that live in salt and fresh water and comprise one of the larger groups of venomous marine animals [1]. The incidence of stingray injury in freshwater is 1.7/100 [2]. Some stingray injuries can be accompanied by infection, toxic effect, and infection by marine bacteria (in 9% of cases) [2-4]. Venom from sea creatures can exacerbate aquatic injuries. Most ocular injuries are conjunctival or corneal abrasions or corneal foreign bodies, and while orbital cellulitis is rare, it is one of the more dangerous complications. We present a case of severe orbital cellulitis resulting from a stingray strike.

## Case presentation

**Figure 1:**
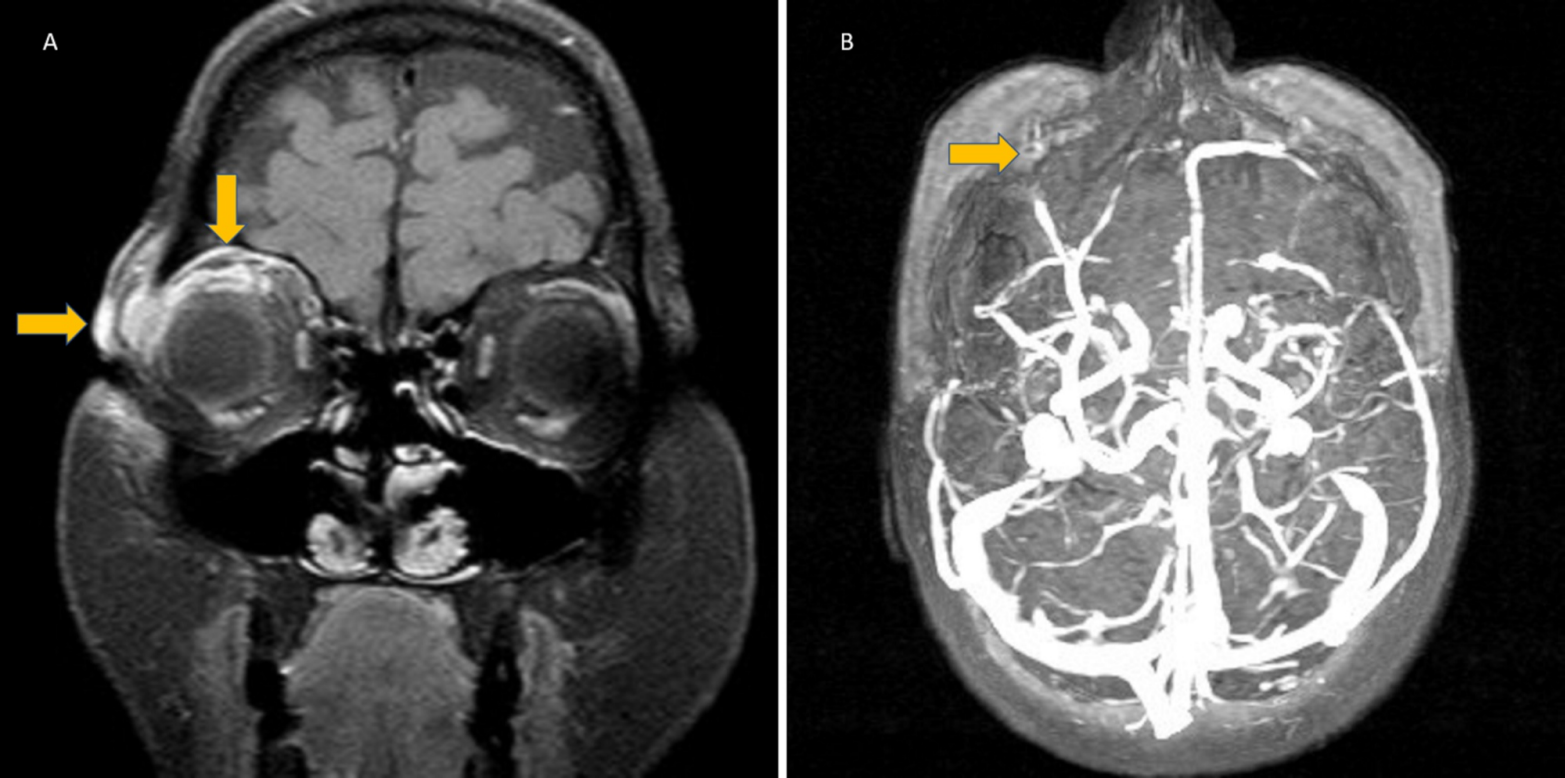
(A) The T1 SPIR contrast enhancement on a coronal MRI slice shows subcutaneous edema, periorbital cellulitis at the right eye. (B) Filling defect due to thrombosis of superior ophthalmic vein on the right eye. SPIR: Spectral presaturation with inversion recovery; MRI: Magnetic resonance imaging.

A 52-year-old woman had a history of travel to Phuket Island. She reported seeing a stingray while staring at the bottom of the sea with her naked eye; the stingray suddenly moved, hitting her right eye with its tail. An ocular dressing was initially placed by a nurse at the beach. She was admitted to the local emergency department when eyelid swelling, ocular pain with movement, diplopia, nausea, and diarrhea developed three hours later. Her initial laboratory test results were as follows: hemoglobin, 13 mg/dl; white blood cell count, 4.3 x 10^3^/mm^3^, platelet count, 247 x 10^3^/mm^3^; and erythrocyte sedimentation rate, 59 mm/hour. Ophthalmic antibiotic drops and oral amoxicillin-clavulanate (2000 mg/day) were administered. She subsequently urgently returned to her home country. She was found to have a corneal injury that was repaired by an ophthalmologist. The patient was then referred to our neurology department for consultation. Ophthalmologic and neurologic examination showed injury to the conjunctiva, corneal abrasion without mention of foreign body, eyelid contusion, and isolated right eye lateral gaze palsy and ptosis. Visual acuity was 20/20, eye pressures were 19 and 15 mmHg in the right and left eye, respectively. Soft tissue swelling with hematoma of the right eyelid and periorbital area including the post-septal area at the lateral extraconal fat of the right orbit were shown on orbital computed tomography. Orbital magnetic resonance (MR) imaging and MR venography showed orbital cellulitis, superior and lateral rectus edema, and thrombosis of the right superior ophthalmic vein (Figure 1A, 1B).

The diagnosis was established as orbital cellulitis and local venous thrombosis. Enoxaparin 6000 IU and prednisolone 60 mg per day were started for six weeks. Her examination showed significant improvement after three months.

## Discussion

Stingray strike injury sites are usually the distal lower extremity. Thoraco-abdominal stingray spine injuries are rare but can cause mortality due to cardiac complications [1]. Stingray injuries can be complicated by poor wound healing, tissue necrosis, and bacteremia. Medical treatment can often involve surgical wound debridement. It has been reported that the incidence of associated infection can be as high as 9% [2]. Orbital cellulitis represents a major infection of orbital tissues. Various patterns of contiguous spread can cause orbital cellulitis such as direct invasion from paranasal infection, direct inoculation from trauma, or hematogenous spread from bacteremia [5].

Generally, stingray strike injury occurs due to deep penetration and retraction of the stingray’s barbed tail spine into the soft tissue, causing physical trauma to the tissues in addition to the physiological response to the spine’s venom, which contributes to widespread tissue damage. Stingray injury can occasionally be accompanied by toxic effects and inoculation by marine bacteria. Such cases are painful and more likely to be associated with septic complications. Empiric antibiotic therapy should be started [6]. Superior ophthalmic vein thrombosis is an uncommon orbital pathology that is frequently associated with cavernous sinus thrombosis. Vascular injury, stasis, and hypercoagulability may cause cerebral vein thrombosis [7,8]. Our patient’s case included these risk factors, and the thrombosis affected the superior orbital vein.

## Conclusions

We report aquatic ocular injury-related orbital cellulitis caused by a stingray attack in a 52-year-old woman. The patient’s orbital cellulitis is believed to have been caused by both the stingray injury and associated marine bacteria inoculation. Physicians should be aware of the consequences of aquatic injuries related to poisonous fish and stingrays.
